# Suppression of IgM Proteolysis by Conformational Stabilization Through Excipients

**DOI:** 10.3797/scipharm.1501-12

**Published:** 2015-03-04

**Authors:** Monika Mueller, Maybelle Q. T. Loh, Pete Gagnon

**Affiliations:** 1 Department of Pharmaceutical Technology and Biopharmaceutics, University of Vienna, Althanstrasse 14, A-1090 Vienna, Austria; 2 Bioprocessing Technology Institute, A*STAR, 20 Biopolis Way, #06-01, Centros, 138668, Singapore

**Keywords:** IgM, Proteolysis, Conformational stability, Sorbitol, Glycine

## Abstract

Protease activity from host cell lines may cause product loss or affect the quality of recombinant proteins. In this study, we showed that excipients like glycine and sorbitol reduce the proteolysis of an immunoglobulin M (IgM) in the presence of added proteases like α-chymotrypsin, papain, and pepsin. The activity of the proteases in the IgM-protective environments was conserved or even enhanced as tested using low molecular weight substrates. Thus, a higher resistance against proteolytic degradation appears to be caused by the conformational stabilization of the IgM due to preferential exclusion of sorbitol and glycine.

## Introduction

Host cell proteases may cause significant product loss or affect the quality of recombinant proteins [1]. Proteases are found in several host cells like cathepsin D-like proteases in hybridoma cells [1], aspartic acid and cysteine proteases like cathepsin L in myeloma cells [2], gelatinase B and cathepsin D in Chinese hamster ovary (CHO) cells [3, 4], or serine and cysteine proteinases in the plant, *Nicotiana benthamiana* [5].

Unfolded or partially folded proteins are more prone to proteolysis than folded proteins as shown previously with ribonuclease S, frataxin, a domain of the glucocorticoid receptor, and the androgen receptor [6–9]. The folded state could be obtained by adding the natural osmolytes [6, 7] or by the binding of a transcription factor [8]. Folded proteins can be further stabilized against proteolysis by adding osmolytes which was shown in trypsinogen, chymotrypsinogen [10], and an IgG [11].

In this study, we investigated the protection of a large, complex glycoprotein, namely an immunoglobulin M (IgM) from proteolysis. IgMs are emerging as therapeutic candidates [12], however, they have the reputation of being labile. We showed previously that IgMs are prone to fragmentation upon storage in phosphate-buffered saline (PBS) [13]. The excipients, sorbitol (20%) and glycine (1 M), protected the IgMs from degradation during accelerated storage at 37°C and long-term storage at 4°C for at least one year [13]. Those excipients stabilize proteins via their preferential exclusion from the protein surface which leads to a layer of excess water surrounding the protein [14, 15]. The protein is forced to minimize its surface area resulting in a more compact, folded state [15].

In the present study, the proteases pepsin, papain, and α-chymotrypsin were added to a purified IgM (mAb 85), and the degradation was analyzed. Furthermore, the mechanism of inhibition of proteolysis was elucidated in the presence and absence of excipients.

## Results and Discussion

IgMs are prone to fragmentation during storage under real time and accelerated stress conditions without excipients [13]. A storage buffer containing 1 M glycine and 20% sorbitol at pH 5.5 suppressed fragmentation from storage at elevated temperatures and under real time storage conditions as shown in a previous study [13]. This raised the question of whether the lack of fragmentation results from the effect of the formulation on the IgM, endogenous proteases, or both. In this study, we show that the resistance of mAb 85 even against proteolysis by added proteases was significantly increased by adding sorbitol and glycine.

**Fig. 1:**
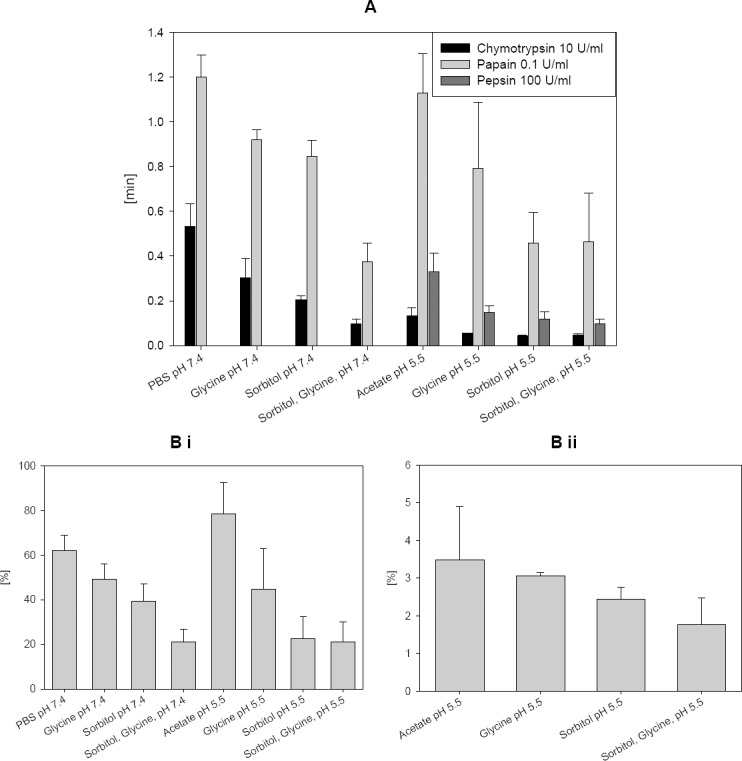
Degradation of mAb 85 by proteases as analysed by SEC: (A) Retention time shift after incubation with α-chymotrypsin, papain, or pepsin; (B) Fragmentation after incubation with the proteases (i) papain and (ii) pepsin. The proteolytic activity of all tested proteases is significantly reduced by adding sorbitol or glycine

mAb 85 was incubated with three different proteases, α-chymotrypsin, papain, and pepsin in the presence and absence of excipients at pH 7.4 and 5.5. The pH optima of the proteases were 7.8 for α-chymotrypsin, 6–7 for papain, and 1.5–2.5 for pepsin, respectively. The retention time shift in the SEC chromatogram, which indicates a decreased molecular mass of degraded monomer, was reduced by adding excipients (Figure 1A). Twenty percent sorbitol showed a higher effect than 1 M glycine. A combination of sorbitol and glycine showed an additive effect for the reduction of the proteolytic cleavage at pH 7.4 and for the protection from degradation by pepsin and chymotrypsin at pH 5.5. For papain at pH 5.5, the degradation in a buffer containing sorbitol and glycine is not significantly different than a buffer containing only sorbitol. In the absence of added proteases, no degradation was found within 2 hours at 37°C, but after 7 days as reported previously [13].

In parallel, the fragmentation percentage is decreased (Figure 1B). Indeed, 0.1 U/ml papain formed 62% fragments in a pH 7.4 buffer which was reduced to 49% by adding glycine, to 39% by adding sorbitol, and to 21% by adding a mixture of both. An amount of 0.1 U/ml papain formed 78% fragments in a pH 5.5 buffer which was reduced to 45% by adding glycine, to 22% by adding sorbitol, and to 21% by adding a mixture of both. Since the protective effect is seen at pH 7.4 and 5.5, the inhibition of fragmentation is not just caused by a pH reduction of the buffer. One hundred U/ml pepsin formed 3.5% fragments in a pH 5.5 buffer which was reduced to 3.1% by adding glycine, to 2.4% by adding sorbitol, and to 1.8% by adding a mixture of both. The fragment percentage after incubation with α-chymotrypsin could not be determined because of an overlay of α-chymotrypsin with the fragments. α-chymotrypsin has a molecular weight of 25 kDa and had to be used in relatively high concentration (0.25 mg/ml) because of its lower activity per mg.

The proteolytic degradation is not totally inhibited, but it has to be considered that the tested concentrations are much higher than concentrations of potentially remaining proteases in purified proteins. The degradation at pH 7.4 and 5.5 was comparable, except for pepsin which is not active at pH 7.4.

This raises the question of how the protection by sorbitol and glycine is occurring. One possibility is the inhibition of the proteases either directly or due to compaction of the protease conformation, which is associated with a lower activity. The other possibility is compaction of the IgM which makes it less accessible for proteolytic cleavage. To elucidate the mechanism, the proteolytic cleavage of low molecular weight substrates was tested. Those substances are not stabilized by compaction of their conformation via the preferential exclusion mechanism. The activity of papain was slightly increased (Table 1A), while the activity of chymotrypsin was virtually unchanged (Table 1B), suggesting that the excipients might stabilize papain without reducing its activity. Such an effect of osmolytes has been described previously for trypsin [10]. These results suspend the possibility that sorbitol and glycine act as inhibitors and suggest that the protective effect is mediated solely through conformational stabilization of mAb 85. This may lead to an increased compactness of proteins and a reduction in the accessibility of the cleavage sites for proteases.

**Tab. 1:**
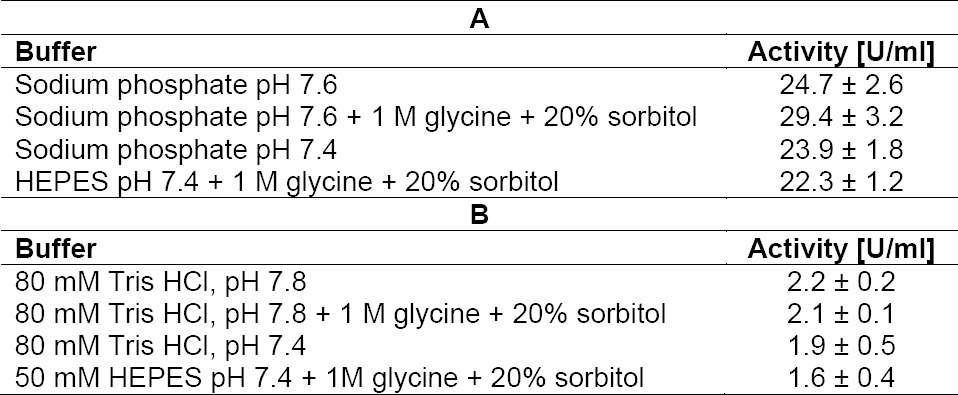
Protease activity tested using low molecular weight substrates: addition of (A) 20 U/ml papain and (B) 2 U/ml chymotrypsin

**Fig. 2:**
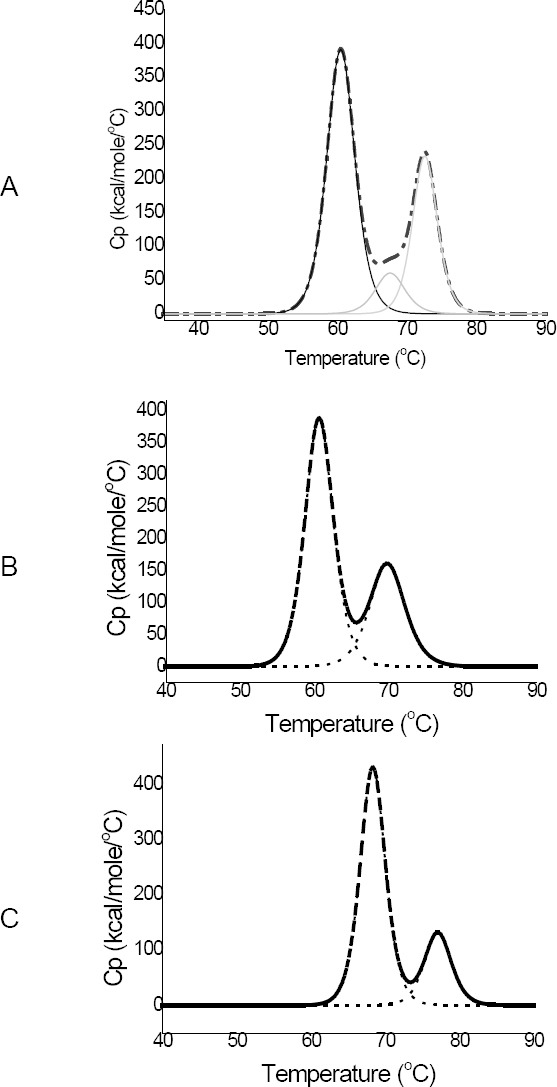
Thermogram of mAb 85 at (A) pH 7.4; (B) at pH 5.5; and (C) at pH 5.5 in the presence of 20% sorbitol and 1 M glycine. mAb 85 unfolds in three transitions at pH 7.4 and in two transitions at pH 5.5

An increase in conformational stability of mAb 85 by glycine and sorbitol was confirmed using DSC (Table 2). mAb 85 unfolded in 2–3 thermal transitions dependent on the pH of the buffer (Figure 2) and corresponded to different domains or structural areas. Due to the lack of data with IgMs, we conclude from data with IgGs that three transitions (like mAb 85 at pH 7.4) correspond to the different unfolding temperatures of Fab, CH2, and CH3 domains and two transitions (like mAb 85 at pH 5.5) correspond to a different unfolding temperature for only Fab and Fc [16, 17]. The T_m_s were significantly increased by adding 20% sorbitol or 1 M glycine (by 4–5°C) or both (by 7–11°C) at pH 5.5 and 7.4 (Table 2, Figure 2). The results are in agreement with previous studies regarding an increase of T_m_ by adding sorbitol and glycine [15, 18]. A correlation between high T_m_ and lower tendency for proteolytic degradation was also reported already [6, 9]. The osmolytes which stabilized proteins against proteolysis in previously published studies were ectoin, hydroxyectoin, prolin, betain [10, 11], trimethylamine N-oxide (TMAO) [7, 8], and sarcosine [6]. They can act via the same stabilization mechanism as sorbitol and glycine, namely preferential exclusion [10, 11]. In contrast to our study, Kolp *et al*. did not find a significant effect of sorbitol up to 15% in comparison with a range of amino compounds [10]. This suggests that preferential exclusion was not the primary stabilizing mechanism in that study, and raises the possibility that protection may have been mediated in part by the homology between those protectants and protease substrates or catabolites. Our experimental design isolated enzyme activity from protein degradation and showed that protection from proteolysis can result solely from conformational stabilization.

**Tab. 2:**
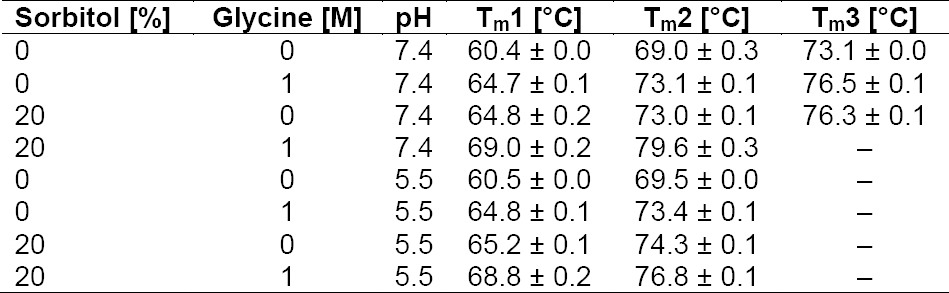
Conformational stability of mAb 85 depending on pH, glycine, and sorbitol concentration as measured by DSC: mAb 85 unfolded in 2–3 thermal transitions (T_m_).

Sorbitol and glycine may also be used to inhibit self-digestion of autoproteolytic proteins. As mentioned previously, the activity of papain in this study and the activity of trypsin in the study of Kolp *et al*. [10] even increased in the presence of excipients. This effect may be caused by the inhibition of autoproteolysis of those enzymes.

Furthermore, sorbitol and glycine may be added already during purification to prevent proteolytic activity. Proteases are often not efficiently removed from recombinant proteins since they can form complexes and stick to the proteins. Protease inhibitors would help to reduce the proteolytic activity, but are not whished in the final therapeutic formulation. Thus, it may be of importance that proteolysis can also be prevented by adding excipients.

## Experimental

### Materials

Glycine, sodium acetate, acetic acid, sodium dihydrogen phosphate monohydrate, disodium hydrogen phosphate, and potassium sulfate were purchased from Merck (Darmstadt, Germany). 4-(2-Hydroxyethyl)piperazine-1-ethanesulfonic acid (HEPES), sorbitol, **Tris(hydroxymethyl)aminomethane hydrochloride (**Tris-HCl), α-chymotrypsin, papain, pepsin, *N*-Benzoyl-L-tyrosine ethyl ester (BTEE), and N-A-Benzoyl-L-arginine ethyl ester hydrochloride (BAEE) were purchased from Sigma–Aldrich (St. Louis, MO, USA). All buffers were filtered using 0.2 µm nitrocellulose membranes prior to usage (Millipore, Carrigtwohill, Ireland).

The mouse monoclonal IgM mAb 85, which targets undifferentiated human embryonic stem cells [19], was produced in hybridomas and purified using polyethylene glycol precipitation and anion exchange chromatography as described previously [20]. Nap-5 columns (GE Healthcare, Uppsala, Sweden) were used to exchange the IgM into the buffers tested in this study.

### Proteolytic Cleavage by Chymotrypsin, Papain, and Pepsin

α-Chymotrypsin (10 U/ml) and papain (0.1 U/ml) were added to mAb 85 (1 mg/ml) in two buffers with different pH, 50 mM acetate buffer, pH 5.5 or 50 mM HEPES buffer, pH 7.4 in the presence and absence of excipients and incubated at 25°C for 2 hours. Incubation with pepsin (100 U/ml) was performed for 2 hours at 37°C. The degradation of mAb 85 was analyzed by size exclusion chromatography (SEC).

### Size Exclusion Chromatography (SEC) with Ultraviolet (UV) Detection

SEC was performed using a Shimadzu LC-10Avp series high-pressure liquid chromatographic system (Shimadzu Corporation, Kyoto, Japan) connected to a TSKgel G4000SW_XL_ column (7.8 mm x 30 cm; Tosoh, Tokyo, Japan). Thirty-five µL of the samples were injected and aggregates, monomers, and fragments were separated using 0.2 M sodium phosphate, 0.1 M potassium sulphate, pH 6.0 buffer, and a flow rate of 0.6 mL/min. The elution profiles were detected at 280 nm. The data was analyzed using Shimadzu Class-VP software (Version 6.14 SP2).

The fragmentation percentages were calculated by dividing the area of the fragment peak by the sum of all peak areas. The retention time shift was related to the retention time of the untreated mAb 85 in the respective buffer.

### Spectrophotometric Assays with Chromogenic Substrates

The activity of α-chymotrypsin was tested using BTEE (0.55 mM) in 38 mM Tris-HCl buffer at pH 7.8 according to the manufacturer’s protocol (Sigma-Aldrich). In brief, the absorbance increase at 256 nm (per minute) was determined and divided by the extinction coefficient of BTEE (0.964). This gives the activity in units/ml since one unit α-chymotrypsin hydrolyses 1.0 µmol BTEE per minute at pH 7.8. The activity of papain was tested using BAEE in 63 mM sodium phosphate buffer at pH 7.6 and according to the manufacturer’s protocol (Sigma-Aldrich). In brief, the absorbance increase at 253 nm (per minute) was determined and divided by 0.001 to obtain the activity in units/ml since one unit of papain caused an absorbance increase of 0.001 per minute at pH 7.6. All assays were also performed at pH 7.4 which was used for mAb 85 cleavage.

### Differential Scanning Calorimetry (DSC)

DSC measurements were performed using the VP-Capillary DSC system (Microcal Inc, Northampton, MA, USA). All samples were analyzed at a concentration of 1 mg/ml using a scan rate of 1°C per minute from 35 to 100°C. Thermograms were corrected by the deduction of corresponding buffer scans and normalized to the protein concentration. Transition curves were fitted using a non-2 state model to determine the thermal transitions midpoint (T_m_) using Origin 7.0 software (OriginLab Corporation, Northampton, MA, USA).

### Statistics

All experiments were performed in triplicate in independent experiments. Data are expressed as the mean ± standard deviation in tables or with error bars in the figures which indicate the standard deviation.

## Conclusion

In conclusion, sorbitol and glycine prevent proteolytic degradation of IgMs by stabilization of their conformation. As IgMs are promising candidates for future therapeutics, it is important to know that they may be stabilized against proteolysis with excipients that may be used in therapeutic formulations.
